# Recent Advances in Fabrication of Flexible, Thermochromic Vanadium Dioxide Films for Smart Windows

**DOI:** 10.3390/nano11102674

**Published:** 2021-10-11

**Authors:** Jongbae Kim, Taejong Paik

**Affiliations:** School of Integrative Engineering, Chung-Ang University, Seoul 06974, Korea; jbkim0406@gmail.com

**Keywords:** VO_2_, phase change material, flexible thin film, thermochromics, energy efficient materials

## Abstract

Monoclinic-phase VO_2_ (VO_2_(M)) has been extensively studied for use in energy-saving smart windows owing to its reversible insulator–metal transition property. At the critical temperature (T_c_ = 68 °C), the insulating VO_2_(M) (space group P21/c) is transformed into metallic rutile VO_2_ (VO_2_(R) space group P42/mnm). VO_2_(M) exhibits high transmittance in the near-infrared (NIR) wavelength; however, the NIR transmittance decreases significantly after phase transition into VO_2_(R) at a higher T_c_, which obstructs the infrared radiation in the solar spectrum and aids in managing the indoor temperature without requiring an external power supply. Recently, the fabrication of flexible thermochromic VO_2_(M) thin films has also attracted considerable attention. These flexible films exhibit considerable potential for practical applications because they can be promptly applied to windows in existing buildings and easily integrated into curved surfaces, such as windshields and other automotive windows. Furthermore, flexible VO_2_(M) thin films fabricated on microscales are potentially applicable in optical actuators and switches. However, most of the existing fabrication methods of phase-pure VO_2_(M) thin films involve chamber-based deposition, which typically require a high-temperature deposition or calcination process. In this case, flexible polymer substrates cannot be used owing to the low-thermal-resistance condition in the process, which limits the utilization of flexible smart windows in several emerging applications. In this review, we focus on recent advances in the fabrication methods of flexible thermochromic VO_2_(M) thin films using vacuum deposition methods and solution-based processes and discuss the optical properties of these flexible VO_2_(M) thin films for potential applications in energy-saving smart windows and several other emerging technologies.

## 1. Introduction

**Figure 1:**
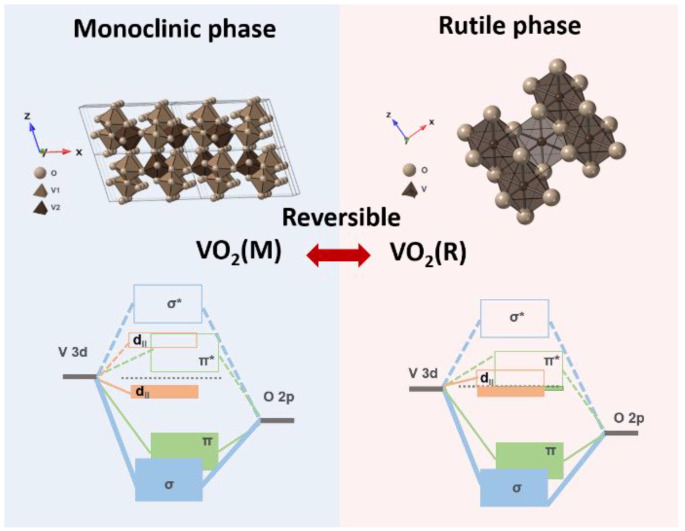
Schematic of the crystal structure and electronic band structure of the insulating VO_2_(M) and the metallic VO_2_(R). Adapted with permission from [13]. Copyright 2011, American Chemical Society.

To address the rapidly increasing energy demand and growing environmental concerns, the development of renewable resources and smart-energy materials is receiving widespread attention [1]. Building energy consumption is estimated to account for 30–40% of the total global energy consumption, and this proportion is expected to continue increasing [2,3]. Windows are the most energy-inefficient component of a building; in this regard, smart windows offer the potential to reduce energy consumption by reducing the air-conditioning load via modulation of solar radiation [4]. Researchers have extensively studied the development of energy-efficient materials for smart windows to address the increasing energy needs. Monoclinic-phase VO_2_ (VO_2_(M)) was first reported by Morin in 1959 and is the most widely studied inorganic material owing to its switchable thermochromic properties [5]. VO_2_ exhibits a first-order insulator–metal phase transition at the critical temperature (T_c_ = 68 °C), accompanied by reversible phase-change properties in the transition from the insulating monoclinic (P21/c) phase to the metallic rutile (P42/mmm) phase [6,7]. Figure 1 shows the crystal structure and band diagram of monoclinic and rutile phase VO_2_. The vanadium ions in the monoclinic phase dimerize to form zigzag atomic chains with two V-V distances of ≈3.12 and ≈2.65 Å. Conversely, in the rutile phase, straight and evenly distanced vanadium chains are formed along the c-axis with ≈2.85 Å of distance and V^4+^ ions surrounded by O^2-^ are located at the center and corner positions [8,9]. Dimerization of the vanadium ion causes the d_ll_ band to split into a filled bonding (d_ll_) and an empty antibonding (d_ll_*). Furthermore, the π* orbitals shift to higher energies and make a forbidden band of approximately 0.7 eV between the d_ll_ and π* [10,11]. The Fermi level is located within the forbidden band, thereby forming the insulating VO_2_(M). When the temperature is higher than T_c_, the density of the Fermi energy states in VO_2_(R) is formed by a mixture of π* and d_ll_ orbitals [9,12]. The electrons at the d_ll_ state exhibit a behavior similar to that of free electrons, accomplishing a half-filled metallic state. The Fermi level form between the π* and d_ll_ bands, indicating an enhanced electrical conductivity of the VO_2_(R) [13]. Therefore, electrical and optical properties are considerably modulated during the phase transition. The phase transition of VO_2_ can be induced by different types of stimuli, such as heat [5], electric fields [14], and mechanical strain [15]. The phase change in VO_2_(M) has also been utilized in various emerging technologies, including optical switches [16], thermoelectrics [17], hydrogen storage [18,19], sensors [20,21,22], transistors [23,24,25], active metamaterials [26,27,28], and photoelectric devices [29]. The application of VO_2_(M) in smart windows was investigated in the 1980s by Jorgenson et al. [7] and Babulanam et al. [30]. When the external temperature is lower than the phase-transition temperature, which is approximately 68 °C, VO_2_(M) exists in the insulating phase, exhibiting high transmittance of near-infrared (NIR) wavelengths in the solar spectrum. Conversely, when the temperature is higher than the phase-transition temperature, the crystal and band structures change because of the transition from the insulator phase (VO_2_(M)) to the metallic phase (rutile VO_2_ (VO_2_(R))), which significantly reduces the optical transmittance of NIR wavelengths. Therefore, thermochromic smart windows can reversibly modulate their solar transmittance at different temperatures and can reduce the room temperature during hot weather conditions; this will reduce the total energy consumption of the building. VO_2_-based thermochromic smart windows offer characteristic advantages over other types of energy-saving windows, such as low-emissivity (low-e) glass [31,32] and electrochromic (EC) windows, owing to their ability to self-regulate solar transmission/reflection according to the external environment without utilizing an external energy supply [33,34,35]. Moreover, thermochromic windows have a relatively simple structure when compared with low-e or EC glass, thereby exhibiting potential for large-area installation and mass production for commercialization [36].

The performance of VO_2_ for smart windows is evaluated in terms of the luminous transmittance (T_lum_) and solar modulation ability. Luminous transmittance refers to the integrated optical amount of visible-light transmittance, which is determined from the following equation:

$$T_{\mathrm{lum}}=\int^{\;}\Phi_{\mathrm{lum}}\left(\lambda \right)\mathrm{Td}\lambda /\int^{\;}\Phi_{\mathrm{lum}}\left(\lambda \right)d\lambda ,\;\left(380\;\mathrm{to}\;780\;\mathrm{nm}\right)$$

T(λ) and Φ_lum_ characterize the transmittance of the wavelength λ and photopic luminous efficiency function in the visible region, respectively [30,37]. Solar-energy modulation ability (ΔT_sol_) is also a critical feature for determining the energy-saving capability of material. ΔT_sol_ is defined as the difference in the solar-energy transmittance (T_sol_) values before and after phase transition in the 240 to 2500 nm spectrum, which is estimated using the follow equations [38]:

$$T_{\mathrm{sol}}=\int^{\;}\Phi_{\mathrm{sol}}\left(\lambda \right)\mathrm{Td}\lambda /\int^{\;}\Phi_{\mathrm{sol}}\left(\lambda \right)d\lambda ,\;\left(250\;\mathrm{to}\;2600\;\mathrm{nm}\right)$$

$$\Delta T_{\mathrm{sol}}=T_{\mathrm{sol},\mathrm{low}\;\mathrm{temperature}}\;-\;T_{\mathrm{sol},\mathrm{high}\;\mathrm{temperature}}$$

where Φ_sol_ denotes the solar irradiance spectrum for an air mass of 1.5, which is equivalent to the presence of the sun at an angle of 37° from the horizon [37]; moreover, T_sol,low temperature_ and T_sol,high temperature_ represent the solar transmittance of VO_2_ films at a low temperature in the monoclinic phase and at a high temperature in the rutile phase, respectively. T_lum_ should be greater than 40% to indicate the requirement for daylight across windows, and ΔT_sol_ should be sufficiently high, at least 10%, for energy saving [39]. Furthermore, the phase-transition temperature of VO_2_ (T_c_ = 68 °C) should be reduced from 68 °C for efficient regulation of solar energy during daytime [40]. Therefore, a reduced phase-transition temperature (T_c_), high luminous transmittance (T_lum_), and strong solar-energy modulation ability (ΔT_sol_) are important characteristics for energy-efficient smart windows. To fulfill the demand for practical applications of energy-saving smart windows, VO_2_-based thermochromic thin films should possess the following features: the phase-transition temperature (T_c_) should be reduced to near-ambient temperature, and a high luminous transmittance (T_lum_ > 40%) accompanied by a strong solar-energy modulation ability (ΔT_sol_ > 10%) should be available [41,42].

Several studies have been conducted to improve the energy-saving performance of VO_2_-based smart windows. For example, reductions in T_c_ have been achieved by doping with metal ions [43,44,45], or by utilizing nonstoichiometric compounds [46], strains [47], and nano-size effects [48]. Among the aforementioned methods, doping with metal ions, such as W^6+^ [49], Al^3+^ [50], Mg^2+^ [51], Sn^4+^ [52], and Mo^6+^ [53,54], is considered the most efficient. However, an increase in the dopant content results in the deterioration of phase-transition behaviors, such as a reduction in ΔT_sol_ and a broadened phase-transition temperature range [55,56]. High values of T_lum_ and ΔT_sol_ are also required to accomplish high-energy modulation efficiency for smart windows; however, these parameters involve a tradeoff, and thus, it is difficult to enhance them simultaneously [57]. Various strategies have been suggested to improve T_lum_ and ΔT_sol_ simultaneously, such as doping with Mg^2+^ [56] and F^−^ [55], or utilizing nano-size thermochromic materials [58], photonic crystals [59], antireflective overcoating [60], porous films [60], and multilayered structures [60,61]. However, the fabrication of VO_2_(M) films with high T_lum_ (> 40%) and ΔT_sol_(>10%) values as well as a sufficiently reduced T_c_ remains challenging, which limits the utilization of VO_2_(M) in practical applications [56,57,62]

Recently, the fabrication of flexible VO_2_(M) films has attracted widespread attention [39,56]. Flexible thermochromic films demonstrate significant potential for large-scale fabrication and commercialization [63,64,65,66]. For example, flexible VO_2_(M) films can be instantly applied to the windows of existing buildings and easily integrated onto curved surfaces, such as automobile windows. Moreover, flexible VO_2_(M) thin films show the potential for application in actuators and optical switches for future optical and electronic devices [63,67]. Thus far, high-quality VO_2_(M) thin films have been fabricated using vacuum-chamber-based techniques, such as chemical vapor deposition (CVD) [68,69,70], physical vapor deposition [56], radiofrequency (RF) magnetron sputtering [71], and pulsed laser deposition [72]. These deposition methods provide high-quality and highly crystalline VO_2_(M) films; however, they often require high-temperature deposition conditions or an additional thermal annealing process to yield phase-pure crystalline VO_2_(M) films [63]. The deposition temperature is typically higher than 400 °C, which exceeds the thermal resistance of most flexible polymeric substrates [51,73,74,75]. Therefore, chamber-based deposition processes are predominantly performed on rigid inorganic substrates with high thermal resistance, which limits the fabrication of crystalline VO_2_(M) films on flexible substrates. Flexible VO_2_(M) films can also be obtained via colloidal deposition using VO_2_(M) nanoparticles (NPs) [56,65,76]. Colloidal dispersion of VO_2_(M) enables solution-based deposition onto polymeric substrates or the formation of flexible composite films through mixing in a polymer matrix. Hydrothermal synthesis of colloidal VO_2_(M) NPs was reported in a recent study, which demonstrated the feasibility of producing flexible VO_2_(M) films through the solution-based deposition of NPs at room temperature [77,78]. However, for colloidal VO_2_(M) NPs synthesized hydrothermally, lowering T_c_ while maintaining favorable optical properties, such as a high T_lum_ and ΔT_sol_, remains difficult [79,80]. Therefore, the fabrication of flexible VO_2_ thin films using colloidal VO_2_(M) NPs with a reduced T_c_, high T_lum_, and high ΔT_sol_ is still significantly challenging. In this review, we focus on the recent advances in the fabrication methods for flexible thermochromic VO_2_(M) thin films. We systematically review the fabrication process, including chamber-based vacuum deposition on flexible substrates that possess high thermal resistance. In addition, we introduce film-transfer techniques used to transfer VO_2_(M) layers deposited on rigid substrates onto flexible polymer substrates. Finally, we introduce the solution-based deposition process using colloidal VO_2_(M) NPs. The optical properties and phase transition behaviors are discussed to investigate the potential of flexible VO_2_(M) films for application in energy-saving smart windows and other emerging technologies. 

## 2. Fabrication Methods

### 2.1. Fabrication of Flexible Monoclinic-Phase VO_2_ (VO_2_(M)) Films via Chamber-Based Deposition

**Figure 2:**
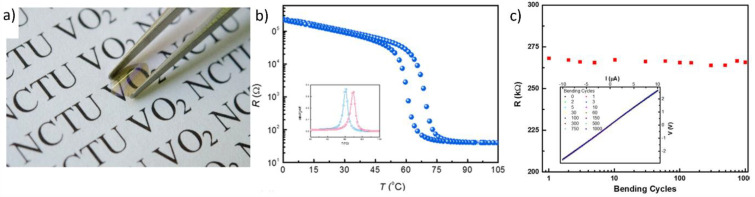
(**a**) Photograph of VO_2_/muscovite thin film; (**b**) Temperature-dependent electrical resistance of VO_2_/muscovite films; (**c**) Cyclability of VO_2_/muscovite films over 1000 iterations in a bending test. Reproduced with permission from [86]. Copyright 2016, American Chemical Society.

As discussed, the fabrication of stoichiometric and highly crystalline VO_2_ films using vacuum deposition requires high-temperature conditions or an additional calcination process [81]. Therefore, rigid inorganic substrates, which have high thermal stability, such as SiO_2_ [82], MgF_2_ [83], and Al_2_O_3_ [84], are generally used for growing VO_2_(M) films. The fabrication of flexible VO_2_(M) films through chamber-based deposition of VO_2_(M) films has also demonstrated using flexible substrates with high thermal stability. For example, muscovite sheets were first used as substrates for the fabrication of VO_2_(M) films because such sheets possess a high thermal stability of over 500 °C and superior chemical resistance, which enable the formation of highly crystalline VO_2_(M) films through high-temperature sintering. High-quality, single-phase VO_2_(M) films can be grown epitaxially on (001) muscovite substrates with high crystallinity, leading to superior phase transition behaviors in terms of resistivity and infrared (IR) transmittance [85]. Li et al. also developed a process for depositing a VO_2_ film directly on a flexible muscovite substrate [86]. First, V_2_O_5_ films were deposited on a native muscovite substrate through pulsed-laser deposition for 20 min; then, the films were annealed at 650 °C under a 5 mTorr oxygen atmosphere to obtain highly crystalline VO_2_(M) films (Figure 2a). The electrical resistance of the VO_2_(M) thin films was measured under various bending radii. During the phase transition, the electrical resistance of the films varied by an order of 10^3^ or more (ΔR/R > 10^3^), and the change in luminous transmittance was higher than 50% (ΔT_r_ > 50%) (Figure 2b). Owing to the intrinsic transparency and flexibility of muscovite sheets, the VO_2_/muscovite heterogeneous structures also exhibited superior flexibility and visible-light transparency. The electrical resistance of the VO_2_/muscovite films remained the same even after the films were bent 1000 times; this confirmed the high mechanical stability of the films (Figure 2c). Thus, considering their enhanced electrical, thermal, optical, and mechanical properties, VO_2_/muscovite films demonstrate considerable potential for application in flexible electronic devices, especially optical switches.

**Figure 3:**
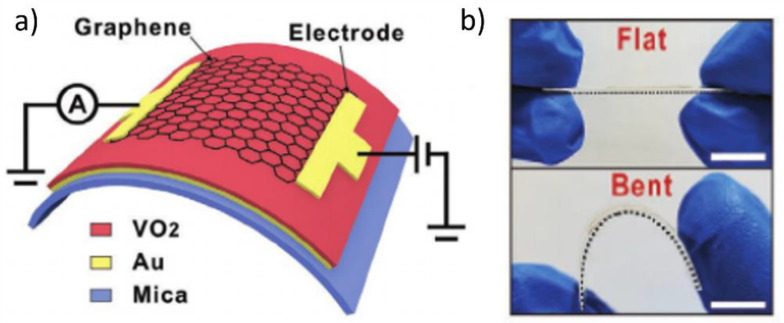
(**a**) Schematic representation of a flexible and electrically tuned flexible phase change material (FPCM) structure; (**b**) Mechanical flexibility of FPCM with 10 mm scale bars. Reproduced with permission from [91]. Copyright 2021, Wiley.

VO_2_(M) thin films grown on substrates, such as TiO_2_, Al_2_O_3_, diamond, and SiO_2_, have strong chemical bonds (ionic or covalent) between the VO_2_(M) layers and the substrates. Thus, the VO_2_ lattice is constrained, which is known as the substrate-clamping effect; this complicates the lattice rearrangement during phase transition [82,87,88]. Therefore, VO_2_ films deposited on inorganic substrates typically require a higher energy to drive the metal–insulator transition (MIT). Conversely, VO_2_(M) films deposited on mica sheets typically have weak van der Waals (vdW) bonds (0.1–10 kJ mol^–1^) between VO_2_(M) and the mica layer, which is 2–3 times weaker than the aforementioned ionic or covalent bonds (100–1000 kJ mol^–1^) [89]. This weak vdW bonding between the VO_2_ film and the mica sheet does not induce any significant lattice strain in the VO_2_ layer. Therefore, the VO_2_ film behaves as a nearly freestanding film on the mica sheet, which enables MIT with exceedingly low energy stimuli [90]. Moreover, owing to the weak vdW bonding between adjacent mica sheets, the thin mica sheet can be peeled off from the substrate, creating transparent and flexible VO_2_(M)/mica sheets. Wang et al. also employed a mica sheet as a support for VO_2_(M) to fabricate a mechanically flexible and electrically tunable flexible phase-change material for IR absorption [91]. First, 100-nm-thick Au thin films were deposited on a mica sheet through magnetron sputtering. Then, a 100-nm-thick vanadium film was deposited on the Au film via electron-beam evaporation and was thermally annealed in an oxygen atmosphere at temperatures of 430–470 °C. Au and mica sheet can withstand high-temperature annealing conditions. Finally, graphene thin films were transferred onto the VO_2_ thin film to deposit the conductive electrode that induces the phase transition of the VO_2_(M) thin films through Joule heating (Figure 3a). The IR absorption of this device can be continuously adjusted from 20% to 90% by changing the current applied to the graphene film. Moreover, this structure exhibited superior bending durability when it was bent up to 1500 times, without any noticeable deterioration in the optical properties (Figure 3b). Such tunable and flexible VO_2_ devices have various application prospects in flexible photodetectors and active wearable devices.

**Figure 4:**
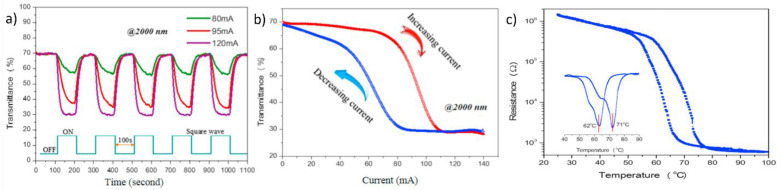
(**a**) Infrared (IR) response of flexible single-walled carbon nanotubes/VO_2_/mica thin film with square-wave current; (**b**) IR performance as a function of applied current (2000 nm); (**c**) Resistance-dependent temperature curve for VO_2_/mica thin film (the inset shows the differential curves during phase transition). Reproduced with permission from [92]. Copyright 2017, Elsevier.

Chen et al. fabricated a flexible VO_2_(M) thin film on a muscovite (mica) sheet directly through RF-plasma-assisted oxide molecular beam epitaxy (rf-OMBE) [92]. First, the VO_2_ layer was grown using rf-OMBE on the (001) plane of mica sheets at 550 °C. Then, a layered single-walled carbon nanotube (SWNT) films was deposited using CVD on the high-quality VO_2_/mica thin film. The SWNT layer exhibited superior conductivity and flexibility and can be employed as an efficient heater when a current/bias voltage is applied. The almost freestanding SWNTs/VO_2_/mica (SVM) film was fabricated by peeling off the thin-layered SVM film from the substrates. Two Au electrodes were deposited on the flexible SVM thin film to provide a two-terminal electrode. The MIT process of the flexible VO_2_(M) thin film can be easily controlled by heating SVM films with a bias current on Au electrodes, thereby enabling reversible modulation of IR transmission. When a bias current was applied, the transmittance decreased sharply from 70% and maintained an almost constant value of approximately 30% thereafter. When the input current was turned off, the transmittance quickly returned to its highest value of 70%; this confirms that direct modulation of the transmittance by applying a current is possible (Figure 4a,b). The MIT temperatures were 71 and 62 °C during the heating and cooling cycles, respectively (Figure 4c). Such ultrathin flexible SVM films with superior flexibility and transparency can be used for various applications involving future electrical devices.

**Figure 5:**
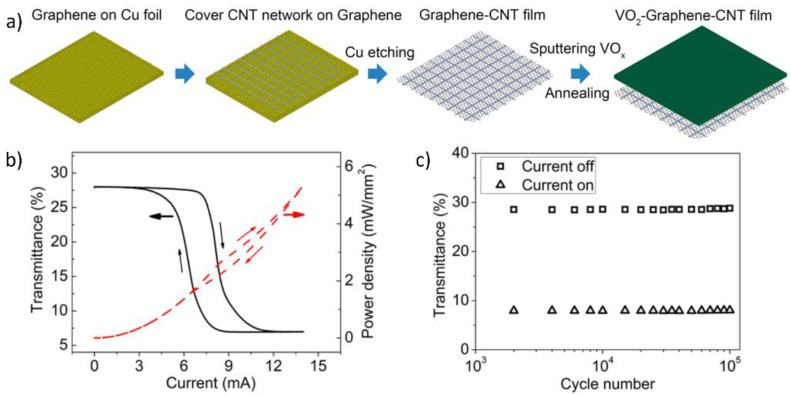
(**a**) Schematic of fabrication of VO_2_/graphene/carbon nanotube (VGC) film; (**b**) Characterization of VGC film with current-dependent transmittance (1500 nm) (black line) and the correlated power consumption (red line); (**c**) Reliability measurement of the VGC films over 100,000 cycles with regard to current pulses. Reproduced with permission from [93]. Copyright 2015, American Chemical Society.

In addition to mica sheets, carbon-based substrates, such as graphene sheets and networks of carbon nanotubes (CNTs), have also been utilized as flexible substrates for VO_2_ deposition owing to their high thermal resistance. Xiao et al. reported the fabrication of VO_2_/graphene/CNT (VGC) flexible thin films [93]. First, the graphene/CNT thin film was prepared by depositing graphene on a Cu substrate via low-pressure CVD. Then, the aligned CNT thin films were stacked on graphene substrates, followed by etching of the Cu substrate to form flexible graphene/CNT flexible thin films. The VO_x_ thin film was deposited on the graphene/CNT film through DC magnetron sputtering and was then thermally annealed at 450 °C in a low-pressure oxygen environment to obtain crystalline VO_2_(M) thin films (Figure 5a). The phase transition of the VGC freestanding thin film can be induced by applying a current. The VGC films exhibited fast switching with low power consumption and highly reliable phase transition (Figure 5b,c). The drastic change in IR transmittance during the phase transition can potentially enable the application of VGC films in IR thermal camouflage, cloaking, and thermal optical modulators.

**Figure 6:**
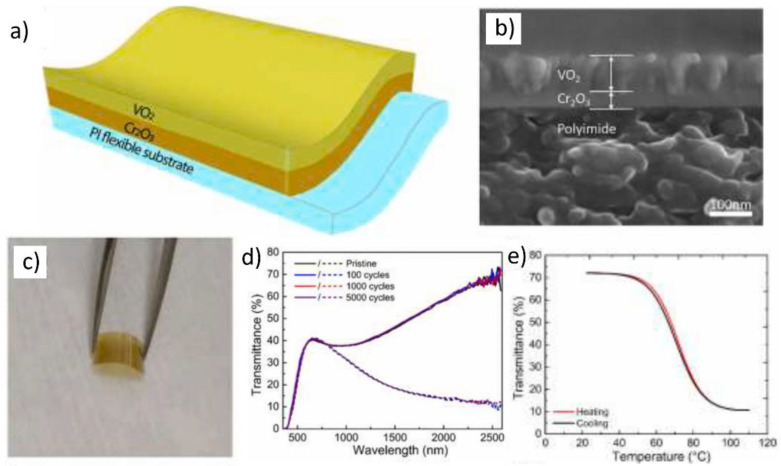
(**a**) Schematic representation and (**b**) cross-sectional scanning electron microscopy image of VO_2_/Cr_2_O_3_/polyimide (PI) film; (**c**) Photograph of flexible VO_2_/Cr_2_O_3_/PI film, (**d**) Ultraviolet–visible–near-IR (NIR) transmittance spectra of flexible VO_2_/Cr_2_O_3_/PI film after multiple bending cycles; (**e**) Temperature-dependent transmittance hysteresis loop (2500 nm) of flexible VO_2_/Cr_2_O_3_/PI film. Reproduced with permission from [94]. Copyright 2021, Elsevier.

Chan et al. reported the fabrication of flexible VO_2_(M)/Cr_2_O_3_/polyimide (PI) films using Cr_2_O_3_ as a buffer layer [94]. The Cr_2_O_3_ layer allows an epitaxial growth of the VO_2_(M) layer, typically at approximately 300 °C, which enables the deposition of VO_2_(M) on the PI polymer substrate at a relatively lower temperature (Figure 6a). The lattice constants for Cr_2_O_3_ are a = 0.496 nm, b = 0.496 nm, and c = 1.359 nm, and those for VO_2_(R) are a = 0.455 nm, b = 0.455 nm, and c = 0.286 nm [95]. Therefore, Cr_2_O_3_ can act as a buffer layer owing to the similarity of its lattice constants with those of VO_2_(R). Therefore, highly crystalline VO_2_/Cr_2_O_3_ films can be successfully fabricated even under relatively low deposition conditions from 250 to 350 °C. Moreover, the refractive index of Cr_2_O_3_ is 2.2–2.3; hence, Cr_2_O_3_ behaves as an antireflective coating on top of the VO_2_(M) layers, leading to a higher optical performance with T_lum_ and ΔT_sol_. The VO_2_ film fabricated at 275 °C showed 42.4% of T_lum_ and 0.4% of ΔT_sol_; in contrast, the VO_2_ film deposited with a 60-nm Cr_2_O_3_ buffer layer exhibits a high ΔT_sol_ value of 6.7% at a similar T_lum_ (43.7%). To fabricate flexible VO_2_/Cr_2_O_3_/PI films, thin Cr_2_O_3_ layers were deposited on colorless PI films through magnetron sputtering; then, the VO_2_ layers were directly deposited on Cr_2_O_3_/PI films using magnetron sputtering (Figure 6b). VO_2_/Cr_2_O_3_/PI films exhibit minimal strain owing to the similar lattice parameters of the two layers. Therefore, flexible VO_2_(M) films have a narrow and sharp hysteresis loop. The VO_2_/Cr_2_O_3_/PI films exhibited superior IR modulation properties, i.e., approximately 60% variation at 2500 nm, when the VO_2_ film thickness was approximately 80 nm (Figure 6c). The T_c_ values of the films calculated during the heating and cooling cycles were 71.8 and 71.3 °C, respectively, and the transition width of the hysteresis loop was approximately 0.5 °C, which is significantly low for a phase transition (Figure 6d,e). Furthermore, the resistivity decreased by more than two orders of magnitude during the phase transition, indicating the high crystallinity of VO_2_(M) films. However, the deposition temperature of >250 °C is still higher than the temperature that typical polymer films can withstand, which limits the utilization of various flexible polymeric substrates other than PI.

**Figure 7:**
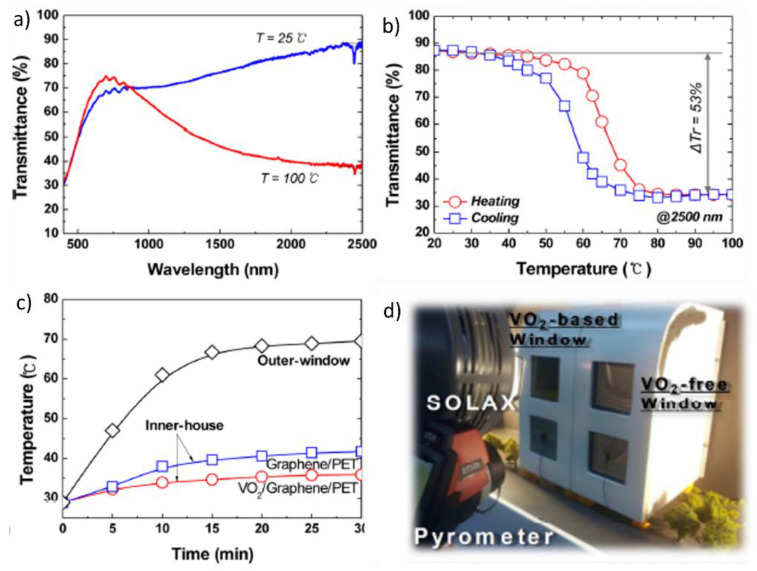
(**a**) Transmission spectra of VO_2_/graphene/polyethylene terephthalate (PET) film at 25 and 100 °C; (**b**) Temperature-dependent transmittance of VO_2_/graphene/PET film at 2500 nm; (**c**) Indoor temperature of a model house with VO_2_/graphene/PET films; (**d**) Photograph of a model house coated with VO_2_/graphene/PET films (VO_2_-based windows) and graphene/PET films (VO_2_-free windows). Reproduced with permission from [98]. Copyright 2013, American Chemical Society.

Although direct deposition of VO_2_(M) on substrates with high thermal resistance is a simple single-step process, only a limited number of substrates can be used under high-temperature deposition conditions. In contrast, the film-transfer process offers opportunities to utilize various types of polymeric substrates for the fabrication of flexible films [96]. In this process, VO_2_(M) films are deposited on rigid substrates via high-temperature vacuum deposition and thermal annealing; then, the VO_2_(M) thin films are transferred onto flexible polymeric substrates using the film-transfer process. As the VO_2_(M) films are deposited under high-temperature conditions, they become highly crystalline, achieving enhanced optical properties (high T_lum_ and ΔT_sol_) and improved stability under ambient conditions that persists for several months [97]. Moreover, polymer supports can impart enhanced mechanical stability and flexibility to films. The fabrication of flexible VO_2_(M) films using the film-transfer process was first performed by Kim et al. [98]. In this process, an atomically thin, flexible graphene film was used to deposit a VO_2_(M) layer for the transfer process. An amorphous VO_x_ layer was first deposited on a graphene/Cu substrate through RF magnetron sputtering. Then, the VO_x_ film on the graphene/Cu substrate was thermally annealed at 500 °C to transform VO_x_ into crystalline VO_2_ films. The Cu substrate was selectively etched, and the remaining VO_2_(M)/graphene film was transferred to a polyethylene terephthalate (PET) film to fabricate flexible VO_2_(M)/graphene/PET films. Because of the deposition on polymer films, the VO_2_(M)/graphene/PET films exhibited high mechanical stability and flexibility while maintaining their reversible phase-transition property. These flexible VO_2_/graphene/PET films exhibited a transmittance of 65.4% at a 550-nm wavelength; moreover, the variation in the transmittance during phase transition reached 53% at a wavelength of 2500 nm, with the transition band width being 9.8 °C (Figure 7a,b). The VO_2_(M)/graphene/PET was integrated onto glass in a model house to investigate its ability to regulate the indoor temperature when functioning as a smart window. The VO_2_/graphene films reduced the indoor room temperature by 5.8 °C compared with bare glass, thereby exhibiting the potential to function as an energy-efficient smart window (Figure 7c,d).

**Figure 8:**
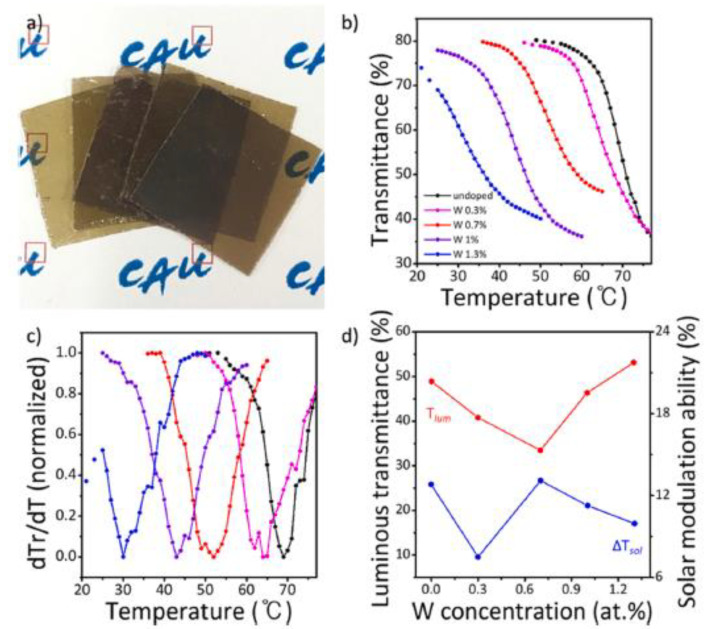
(**a**) Photograph of VO_2_(M) thin films on PET substrates for various W doping concentrations; (**b**) Temperature dependence of transmittance; (**c**) First derivatives of transmittance; (**d**) Luminous transmittance (T_lum_) and solar modulation ability (ΔT_sol_) of VO_2_(M)/mica thin films under various W doping concentrations (1900 nm). Reproduced with permission from [74]. Copyright 2021, Elsevier.

The fabrication of flexible VO_2_(M) thin films with a reduced T_c_ is still challenging owing to the difficulty in doping during the deposition process. Chae et al. reported a solution-based process to deposit VO_2_(M) films using W-doped colloidal NPs, followed by film transfer, to fabricate flexible W-doped VO_2_(M) films [74]. Colloidal VO_x_ NPs were synthesized via high-temperature thermal decomposition with vanadium precursors, which were used for the deposition of VO_2_(M) layers [99]. During the synthesis, W precursors were added into the reaction mixture for efficient doping of W during the formation of VO_x_ NPs. Then, VO_x_ NPs were deposited on mica substrates using the solution-based process and thermally annealed to form highly crystalline VO_2_(M) films. Subsequently, the VO_2_(M)/mica films were transferred onto polymeric substrates using adhesive-coated PET films. During the transfer process, a thin layer of mica sheet was peeled off and transferred to the polymer film to form transparent and flexible mica/VO_2_(M)/PET films. As mica sheets are brittle, the polymer substrate can provide high mechanical strength and ensure reliable bending (Figure 8a). The W dopants were effectively doped into the VO_2_(M) thin films, which resulted in the systematic reduction in T_c_ depending on the different possible doping concentrations. The T_c_ of flexible mica/VO_2_(M)/PET films can be easily controlled at 25.6 °C when 1 at% W doping is used (Figure 8b). These flexible films exhibit superior optical properties—a T_lum_ of 53% and a ΔT_sol_ of 10%—at a T_c_ of 29 °C when 1.3 at% Tungsten (W) doping is used. Thus, such films can be viable for use in energy-saving smart windows (Figure 8c,d).

### 2.2. Fabrication of Flexible VO_2_(M) Films through Solution-Based Deposition Process

Although the vacuum chamber-based deposition and film-transfer processes are highly effective for the fabrication of crystalline VO_2_(M) films on flexible substrates, these processes are significantly complex, involving multiple deposition steps and often requiring an etching process, which can potentially limit large-scale fabrication and commercialization [100]. In contrast, the solution-based process enables simple, low-cost, and large-area fabrication of flexible VO_2_(M) films [101]. Early examples of solution-processed VO_2_(M) films were demonstrated via a sol–gel process [102]. Speck et al. were the first to demonstrate the sol–gel deposition of VO_2_(M) films using molecular vanadium precursors [103]. In general, the sol–gel process of VO_2_(M) thin films have been performed on thermally stable substrates, such as quartz, mica, or silicon wafers, owing to the high temperature thermal annealing process, typically above 400 °C [104]. Recent literature demonstrates that low temperature sol–gel deposition of VO_2_(M) film processes can be achieved using deep ultraviolet photoactivation chemistry, which enable the fabrication of flexible VO_2_(M)/Al_2_O_3_/PI films at 250 °C [105]. Not only has the sol–gel process been widely studied for the fabrication of flexible smart windows, but solution-based deposition using colloidal VO_2_(M) NPs has too. Among a variety of synthetic methods based on colloidal VO_2_(M) NPs, hydrothermal synthesis has attracted considerable attention owing to the high phase purity of the as-synthesized VO_2_(M) NPs [38]. Hydrothermal synthesis involves a chemical reaction that yields high-quality crystals in a sealed pressurized reactor under high pressure and temperature. Hydrothermal growth of VO_2_(M) films on the substrates has also been reported in the literature [106,107]. For example, VO_2_(M) films have been fabricated via hydrothermal reactions by placing r-Al_2_O_3_ substrates in a hydrothermal reactor containing a solution mixture of ammonium metavanadate and oxalic acid [108]. The self-organized VO_2_(M) films were formed with T_lum_ of 65% and ΔT_sol_ of ~11.82% [109]. However, for the direct hydrothermal deposition of VO_2_(M) films on flexible substrates, the substrates should have high thermal and chemical resistance to ensure that they can withstand hydrothermal reaction conditions and calcination temperature [110,111,112]. Therefore, the use of VO_2_(M) NPs for film depositions could have potential for large-area fabrication by mass-production processes using various substrate types. The single-step hydrothermal synthesis of VO_2_(M) NPs was first demonstrated by Théobald et al. using a V_2_O_3_–V_2_O_5_–H_2_O system, and the reaction was performed at a temperature of 20–400 °C under supercritical pressure [113]. There exist several stable vanadium oxide structures, such as VO_2_, V_2_O_5_, V_2_O_3_, V_5_O_9_, V_6_O_13_, and V_6_O_11_, with various nonstoichiometric compounds [114]. Even in the stoichiometric compound, i.e., VO_2_, several polymorphs exist, such as VO_2_(A) [115], VO_2_(B) [59], VO_2_(D) [116], VO_2_(P) [117], and VO_2_(M) [118]. Therefore, hydrothermal synthesis of phase-pure and highly crystalline VO_2_(M) is significantly challenging. Strong phase transition behaviors and favorable optical properties, including high values of T_lum_ and ΔT_sol_, can be obtained using high-purity VO_2_(M) NPs, in the absence of nonstoichiometry and impurities of metastable polymorphs. Therefore, careful control of synthetic procedures, including the hydrothermal reaction conditions, types of metal precursors, solvents, and additives, is a prerequisite for obtaining phase-pure VO_2_(M) NPs.

To enhance phase purity and crystallinity, a two-step hydrothermal synthesis process to synthesize VO_2_(M) NPs has widely studied. In this process, metastable VO_2_ NPs are first synthesized hydrothermally and then thermally annealed for the conversion into the VO_2_(M) phase. Phase-pure VO_2_(M) NPs are obtained from various types of metastable VO_2_ NPs and under different annealing conditions. Xie et al. first reported the hydrothermal synthesis of VO_2_(D) with a size of 1–2 μm, using NH_4_VO_3_ and H_2_C_2_O_4_. Hydrothermal synthesis was performed at 210 °C for 24 h, followed by a calcination process to transform the VO_2_(D) into VO_2_(M) [116]. Calcination of VO_2_(D) was performed at temperatures as low as 300 °C for 2 h under a flow of high-purity nitrogen to obtain VO_2_(R) NPs. These NPs also exhibit MIT near 68 °C. A two-step hydrothermal synthesis using VO_2_(B) NPs has also been reported; however, the phase transformation from VO_2_(B) to VO_2_(M) occurs at a significantly higher annealing temperature, typically higher than 500 °C [119]. Corr et al. also studied the hydrothermal synthesis of VO_2_(B) nanorods using V_2_O_5_ and formaldehyde solution at 180 °C for two days [120]. Then, thermal annealing was performed to convert VO_2_(B) to VO_2_(R) at 700 °C for 1 h in an argon atmosphere. Sun et al. reported the hydrothermal synthesis of VO_2_(P) using VO(OC_3_H_7_)_3_ and oleylamine at 220 °C for 48 h; then, they obtained VO_2_(M) after thermal annealing at 400 °C for 40 or 60 s in a nitrogen or air atmosphere [121]. The size-dependent MIT property of VO_2_(M) NPs was demonstrated through in situ variable-temperature IR spectroscopy. The authors observed that the variation in the transmittance of single-domain VO_2_(M) NPs during phase transition systematically increased with a reduction in the size of the VO_2_(M) NPs. Zhong et al. reported star-shaped VO_2_(M) NPs that were hydrothermally synthesized using NH_4_VO_3_ and formic acid for two days at 200 °C. Then, the as-synthesized NPs were thermally annealed at 300–450 °C for 1 h to obtain VO_2_(M) NPs. The VO_2_(M) NP thin films were 325 nm thick and exhibited a T_lum_ and ΔT_sol_ of 44.18% and 7.32%, respectively [122]. Song et al. reported the hydrothermal synthesis of VO_2_(D) using NH_4_VO_3_ and H_2_C_2_O_4_·2H_2_O at ~220 °C for ~18 h, followed by thermal annealing of VO_2_(D) at 250–600 °C for 3 h, to obtain VO_2_(M) nanoaggregates [123]. The as-synthesized VO_2_(M) exhibited a low T_c_ of approximately 41.0 °C and a thermal hysteresis width of approximately 6.6 °C. Li et al. demonstrated the electrothermochromicity of VO_2_(M) NPs/Ag nanowire (NW) thin films deposited on glass and flexible PET substrates [124]. VO_2_(M) NPs were hydrothermally synthesized using V_2_O_5_ and an oxalic acid dehydrate via at 220 °C for 36 h, followed by additional thermal annealing at 400 °C for 1 h in a vacuum chamber. The VO_2_(M) NPs were deposited on top of Ag NW heaters. The optical response of the VO_2_(M) NP films was then dynamically modulated by applying voltage on Ag NW. The infrared (IR) transmittance variation of the films from 0 V to 8 V of applied voltage is approximately 50% at 1500 nm. Li et al. demonstrated the two-step hydrothermal synthesis of VO_2_(M) NPs using V_2_O_5_, H_2_C_2_O_4_, and polyvinyl alcohol precursors [125]. The hydrothermal synthesis was performed at 220 °C for over 36 h, and the calcination was performed at 300–450 °C under vacuum. The VO_2_(M) film had a thickness of 463 nm and exhibited a high T_lum_ of over 70% at 700 nm; moreover, its IR transmittances at 1500 nm were approximately 89.5% and 53.8% before and after phase transition, respectively. The IR modulation exceeded 35%, which represents favorable optical properties for application in smart windows.

**Figure 9:**
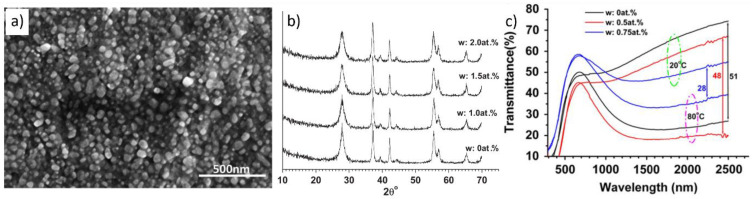
(**a**) SEM image and (**b**) XRD patterns of VO_2_(M) NPs; (**c**) Temperature-dependent transmittance spectra during phase transition of VO_2_(M) film. Reproduced with permission from [134]. Copyright 2011, Elsevier.

A single-step hydrothermal synthesis without a calcination process has also been reported. This method is a potentially simple, convenient, and low-cost process because it involves no additional post-annealing to obtain phase-pure VO_2_(M) NPs [126]. An additional thermal annealing processes induces grain growth in VO_2_(M) films. Size dependence of VO_2_(M) NPs on thermochromic properties have also been reported. Notably, a decrease in the size of VO_2_(M) NPs improves T_lum_ and ΔT_sol_ values [80]. Narayan et al. reported a phase-transition model in which the hysteresis width is directly proportional to the grain boundary area per unit volume [127]. Therefore, the hysteresis width is inversely correlated to the particle radius, and as the particle size increases, the phase transition temperature reduces, and the hysteresis width decreases. The smaller the nanoparticle size, the wider the hysteresis, and the VO_2_ thermochromic performance is improved [128,129]. Therefore, single-step hydrothermal synthesis is more preferable to prevent particle coarsening by an additional thermal annealing process, hence sustaining a high thermochromic performance [130,131]. Gao et al. first demonstrated single-step hydrothermal synthesis of W-doped snowflake-shaped VO_2_(R) using V_2_O_5_ and H_2_C_2_O_4_. The reaction was performed for seven days at 240 °C, and VO_2_(M) NPs were synthesized without a thermal annealing step [78]. The width of the VO_2_(R) nanocomposites was 200–300 nm, and the thickness was approximately 200–800 nm. Alie et al. also demonstrated single-step hydrothermal synthesis of star-shaped and spherical VO_2_(M) particles using H_2_C_2_O_4_ and V_2_O_5_ in a molar ratio of 3:1 at 260 °C for 24 h [132]. The highly crystalline star-shaped VO_2_(M) particles exhibited a high thermal stability of up to ~300 °C and a >10% transmittance variation in the IR region during phase transition. Li et al. reported one-step hydrothermal synthesis of VO_2_(M) NPs using V_2_O_5_, TiO_2_, and H_2_C_2_O_4_∙2H_2_O at 240 °C for 24 h [133]. The VO_2_(M) NPs, with a size of approximately 50–100 nm, were further modified using Zn(CH_3_COO)_2_ to obtain a VO_2_–ZnO structure. The VO_2_(M)–ZnO films exhibited a low T_c_ of approximately 62.6 °C and a T_lum_ and ΔT_sol_ of approximately 52.2% and 9.3%, respectively. Ji et al. demonstrated the synthesis of VO_2_(M) using V_2_O_5_, N_2_H_4_, and H_2_O_2_ through a one-step hydrothermal process performed at 260 °C for 24 h (Figure 9a,b). The as-prepared VO_2_(M) NPs exhibited a transmittance change of approximately 50% at a wavelength of 2000 nm [134]. Moreover, as the concentration of the W dopant increased from 0% to 1%, the T_c_ of the VO_2_(M) NPs decreased from 55.5 to 37.1 °C (Figure 9c). Chen et al. reported the synthesis of phase-pure V_1-x_W_x_O_2_ nanorods using H_2_C_2_O_4_∙2H_2_O and V_2_O_5_ precursors. For W doping, (NH_4_)_5_H_5_[H_2_(WO_4_)_6_]H_2_O was added, and the reaction was performed at 260–280 °C for 6–72 h [135]. The T_lum_ of the 0.5 at% W-doped VO_2_(M) films was 60.6% at 20 °C, and ΔT_sol_ was 8.1%. Whittaker et al. reported the synthesis of W-doped VO_2_(M) nanobelts using V_2_O_5_ and H_2_C_2_O_4_ precursors with H_2_WO_4_ for W doping [43]. The reaction was performed at 250 °C for 12 h to 7 days. W doping (0.90%) led to remarkable modulation of the T_c_ of VO_2_(M) films, from 68.0 to 33.8 °C. Shen et al. demonstrated that Zr doping significantly enhances optical properties while reducing T_c_. [118]. Moreover, Zr doping of VO_2_(M) reduces T_c_ while improving T_lum_ and ΔT_sol_. However, T_c_ is only reduced from 68.6 to 64.3 °C with 9.8% Zr doping; conversely, Zr-doped VO_2_ flexible films exhibit high values of T_lum_ (60.4%) and T_sol_ (14.1%). The optical bandgap, which is 1.59 eV for undoped VO_2_(M), increases to 1.89 eV after 9.8% Zr doping, resulting in a change in the apparent color of the VO_2_(M) films. Accordingly, the color of the Zr-doped VO_2_(M) flexible films is affected; the brown-yellow color of flexible VO_2_(M) film is brightened, along with an increase in T_lum_. In addition, T_c_ is further reduced to 28.6 °C, and T_lum_ and ΔT_sol_ values of 48.6% and 4.9%, respectively, are achieved through W-Zr-co-doping.

**Figure 10:**
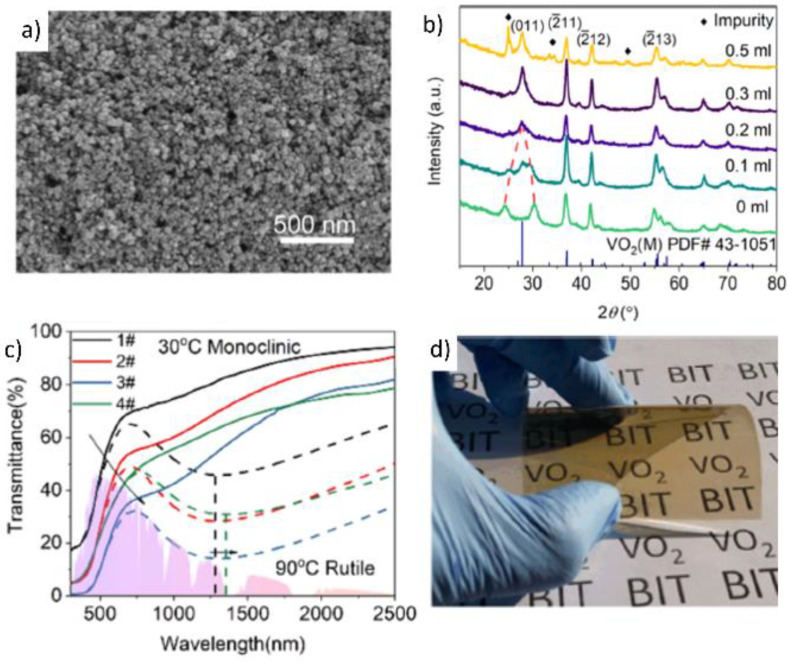
(**a**) SEM image of the VO_2_ NPs with 0.2 mL of H_2_O_2_, (**b**) XRD patterns of as-synthesis VO_2_ NPs with different amounts of H_2_O_2_, (**c**) temperature-dependent transmittance spectra of samples at 30 °C (bold line) and 90 °C (dashed line), and (**d**) the VO_2_(M) NPs-based flexible films. Reproduced with permission from [77]. Copyright 2018, American Chemical Society.

Several studies have been conducted to optimize the conditions for single-step hydrothermal synthesis to enhance the phase purity of VO_2_(M) NPs and their optical properties, including T_lum_ and ΔT_sol_. Guo et al. performed a one-step hydrothermal synthesis process using VOSO_4_ and N_2_H_4_·H_2_O in the presence of H_2_O_2_ [77]. H_2_O_2_, a strong oxidizing agent, is separated after the reaction with the vanadium solution in a hydrothermal autoclave reactor. Then, H_2_O_2_ decomposes and evaporates at 150 °C to provide a moderately oxidizing environment. This facilitates the synthesis of stoichiometric and highly crystalline VO_2_(M) NPs. The as-synthesized VO_2_(M) NPs exhibited an average size of ~30 nm, with significant size uniformity (Figure 10a,b). For the preparation of flexible VO_2_(M) films, the VO_2_(M) NPs were dispersed in N,N-dimethylformamide with polyacrylonitrile polymers. Then, the solution was deposited on a flexible PET substrate. The flexible VO_2_(M) films attained favorable optical properties, with a T_lum_ of 54.26% and a ΔT_sol_ of 12.34% (Figure 10c,d). In addition to optimizing the hydrothermal reaction conditions, the enhancement in the purity of vanadium precursors also produces VO_2_(M) NPs with improved optical properties.

**Figure 11:**
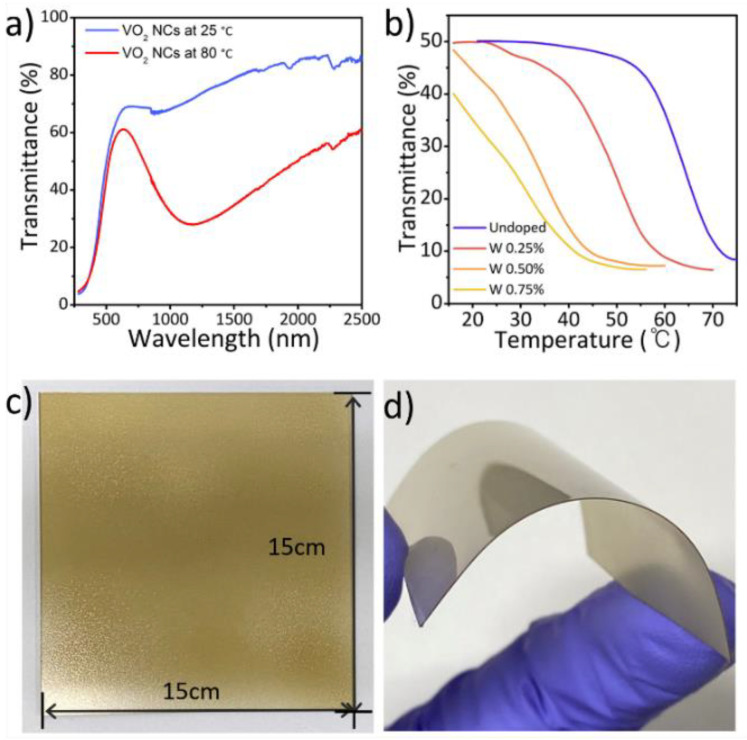
(**a**) Transmittance spectra of VO_2_(M) NP films before and after phase transition; (**b**) Temperature-dependent transmittance (1350 nm) of W-doped VO_2_(M) NP films during heating; Photographs of (**c**) 15 cm × 15 cm VO_2_(M) NP films on glass substrate and (**d**) flexible substrate obtained via spray-coating. Reproduced with permission from [136]. Copyright 2021, Elsevier.

Kim et al. demonstrated single-step hydrothermal synthesis of VO_2_(M) NPs using phase-pure vanadium precursors [136]. After mixing the vanadium precursors, size-selective purification was performed to enhance the phase purity of the precursors, resulting in the formation of VO_2_(M) NPs with enhanced optical properties. The obtained phase-pure VO_2_(M) NPs exhibited an enhanced T_lum_ (55%) and ΔT_sol_ (18%), and the ΔT_sol_ value is one of the highest reported for hydrothermally synthesized VO_2_(M). Furthermore, W-doped VO_2_(M) NPs have been reported to exhibit superior phase-transition behaviors, while T_c_ is systematically reduced depending on the W doping concentration (Figure 11a,b). Flexible VO_2_(M) films were fabricated and deposited on PET polymer substrates over a large area using a spray coater (15 cm × 15 cm) (Figure 11c,d). In model house experiments under daytime solar irradiation, the W-doped VO_2_(M) films applied onto glass provided a significant reduction in the indoor temperature; thus, these films are potentially viable for practical applications.

**Figure 12:**
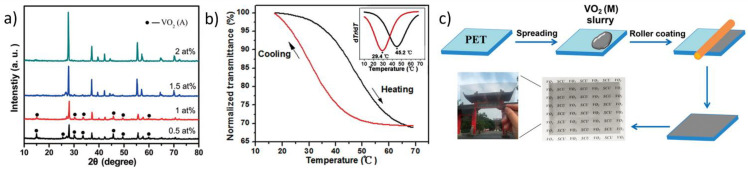
(**a**) XRD patterns of W doped VO_2_(M) films, (**b**) Transmittance hysteresis loops and first derivatives of transmittance for W doped VO_2_ (M) films recorded at a wavelength of 9 μm, (**c**) Schematic diagram of film deposition with W doped VO_2_(M) NPs on PET substrates. Reproduced with permission from [49]. Copyright 2016, American Chemical Society.

Colloidal NPs enable convenient, large-scale fabrication of flexible VO_2_(M) film through the solution process, which is beneficial considering the expected requirement for large-scale fabrication techniques [63,65,76,137]. For the fabrication of flexible VO_2_(M) films, VO_2_(M) NPs have been coated on flexible polymer films or embedded into a polymer matrix [138]. Shen et al. reported a process for blade coating of VO_2_(M) NPs on indium tin oxide (ITO)-coated PET substrates to form flexible VO_2_(M) films [139]. Applying a current along the ITO layer induced ohmic heating, which resulted in the phase transition of VO_2_(M) layers and a change in IR transmittance. The obtained film showed well-controlled IR switching properties upon changing the input voltage, as well as superior thermochromic properties (T_lum_ of 57.3% and ΔT_sol_ of 13.8%). Under ohmic heating, the IR conversion properties did not show any evident deterioration, even after 10,000 bending cycles, which indicates superior stability and flexibility. Chen et al. demonstrated the preparation of VO_2_(M)/polymer composite films by embedding VO_2_(M) NPs into a polymeric matrix. VO_2_(M) NPs were synthesized hydrothermally using V_2_O_5_ and N_2_H_4_ at approximately 180–400 °C for 15 h [80]. The size of the VO_2_(M) NPs ranged from approximately 25 to 45 nm. The synthesized VO_2_(M) NPs were dispersed in polyurethane (PU) and coated onto PET to form flexible VO_2_(M) films. These films achieved high optical performance, with a ΔT_sol_ of 22.3% and a T_lum_ of 45.6%. Similarly, Zhou et al. reported the roll-coating of Mg-doped VO_2_(M) NPs that were hydrothermally synthesized using V_2_O_5_ and H_2_C_2_O_4_ [140]. Mg-doped VO_2_(M) composite foils were prepared by mixing NPs with PU solutions and were deposited on a PET substrate using a roll-coater. The flexible composite foils exhibited a high T_lum_ and ΔT_sol_ of 54.2% and 10.6%, respectively. Liang et al. reported the bar-coating of W-doped VO_2_(M) nanorods; the nanorods were prepared via one-step hydrothermal synthesis for 48 h at 240 °C using V_2_O_5_, C_4_H_6_O_6_, and ammonium tungstate (Figure 12a,b) [49]. The nanorods were then mixed with the tetraethyl orthosilicate and poly(ethyl methacrylate) solution. The solution mixture was cast on PET substrates using a stainless-steel coating bar to fabricate large-area, flexible VO_2_(M) films (Figure 12c). The T_c_ of the flexible VO_2_(M) films could be systematically modulated by approximately 24.52 °C for 1 at% of W doping, and the mid-infrared transmission could be modulated by 31% at a T_c_ of 37.3 °C. Inkjet printing has also been widely utilized as a useful direct-write technology to fabricate high-resolution, low-cost, large-area, and uniform-surface films on flexible substrates [141,142]. Haining et al. reported the fabrication of VO_2_(M) smart windows via inkjet printing using hydrothermally synthesized VO_2_(M) NPs [143,144]. Large-area VO_2_(M) films were fabricated on polyethylene substrates with a T_lum_ of 56.96% and a ΔT_sol_ of 5.21%.

The chemical instability of VO_2_(M) NPs can potentially limit their long-term usage as smart windows in real-world environments [145]. To enhance the chemical stability of VO_2_(M) NPs, core–shell structures, in which VO_2_(M) NPs are overcoated with chemically inert shells, have been developed. Gao et al. reported a core–shell structure with VO_2_@SiO_2_ NPs. VO_2_(M) was synthesized through a hydrothermal reaction, and SiO_2_ shells were overcoated using the Stöber method [56]. SiO_2_ is chemically inert and optically transparent, which is ideal for protecting VO_2_(M) NPs. VO_2_@SiO_2_ NPs exhibit improved chemical resistance to oxidation. The SiO_2_ shell of VO_2_ NPs serves as an oxygen diffusion barrier layer, which can prevent the VO_2_ from changing to V_2_O_5_. This phenomenon was confirmed through experiments conducted with VO_2_ NPs and VO_2_@SiO_2_ NPs after annealing in an air atmosphere for 2 h at 300 °C. Flexible films were fabricated by embedding VO_2_@SiO_2_ NPs into a PU matrix; then, the VO_2_@SiO_2_ NPs/PU cast on a PET matrix were dispersed to fabricate flexible VO_2_@SiO_2_/PU composite films. These films exhibited a high T_lum_ (55.3%) and ΔT_sol_ (7.5%). In addition to SiO_2_, various types of oxides, such as ZnS [146], TiO_2_ [11], and ZrO_2_ [147], have been utilized for overcoating to prepare core–shell NPs. Saini et al. demonstrated an approach to improve the thermal stability and thermochromic properties of VO_2_(M) NPs by overcoating with CeO_2_ [148]. VO_2_(M)@CeO_2_ NPs were observed to be thermally stable for up to 320 °C in air, which confirmed the enhancement in stability after overcoating.

## 3. Perspectives

**Figure 13:**
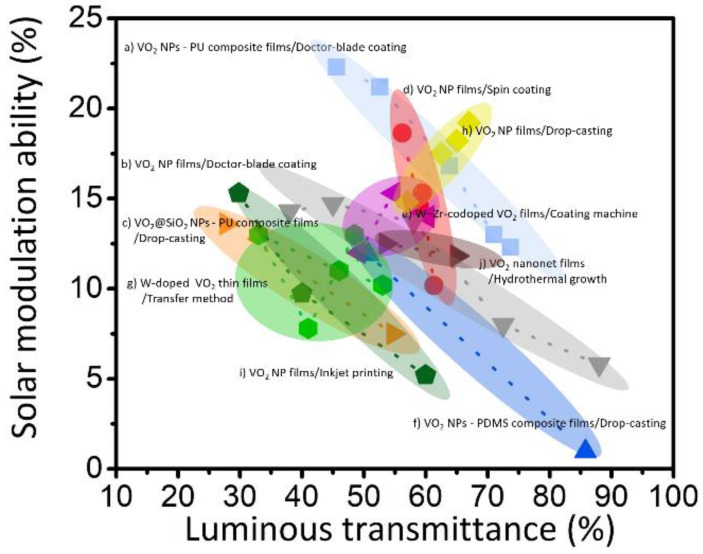
Comparison of luminous transmittance (T_lum_) and solar modulation ability (ΔT_sol_) between flexible VO_2_ thin films fabricated via hydrothermal reaction: (a) [80], (b) [139], (c) [56], (d) [136], (e) [118], (f) [149], (g) [74], (h), [64], (i) [143], (j) [109].

Flexible VO_2_(M) films offer significant potential for the integration of energy-saving smart windows in existing buildings, as well as for application in novel flexile devices, such as sensors and actuators. Various methods for fabricating flexible thermochromic thin films based on the vacuum deposition and solution-based process have been reported; these methods are potentially suitable for commercialization. However, certain issues still remain to be resolved before VO_2_-based smart windows can be utilized in practice. For example, flexible VO_2_ films fabricated using vacuum deposition and film-transfer techniques show high T_lum_ and ∆T_sol_ values; however, these methods are still limited in terms of large-area and mass-production capabilities. In addition, deposition methods with uniform doping should be developed further to systematically reduce T_c_ while maintaining favorable phase-change optical properties. Conversely, the annealing-free, solution-based process offers advantages such as convenient, low-cost, large-area deposition of phase-change VO_2_(M) on flexible substrates. Particularly, hydrothermal synthesis yields highly crystalline VO_2_(M) NPs with colloidal stability and moderately useful phase-change behaviors. However, it is still challenging to prepare flexible VO_2_(M) films with high T_lum_ and ∆T_sol_ values as well as a reduced T_c_. The optical properties of the representative flexible VO_2_ films fabricated using deposition and solution-based processes are summarized in Figure 13, which displays the opportunities for utilizing flexible VO_2_(M) films in energy-saving smart windows. Therefore, large-scale, high-throughput, mass-production capabilities for the fabrication and commercialization of high-performance VO_2_(M) films should be realized. Finally, certain limitations in terms of the intrinsic properties of VO_2_(M) should be overcome to utilize flexible VO_2_ films. First, phase-change VO_2_ films show an inherent brown color, which is not desirable for window applications. Therefore, it is highly recommended to develop fabrication methods that can enable control of the apparent colors of VO_2_(M) while ensuring a high T_lum_ and ∆T_sol_ and low T_c_. Moreover, vanadium oxide has various stable phases and a stable stoichiometry; consequently, VO_2_(M) films are easily oxidized into other phases under exposure in ambient conditions. Therefore, processes to prevent VO_2_(M) from being oxidized, for example, overcoating of VO_2_(M) films or using NPs with protective layers, should be developed to enable long-term usage of the films.

## Data Availability

The data presented in this study are available on request from the corresponding author.
